# Campbell title registrations to date – July 2021

**DOI:** 10.1002/cl2.1192

**Published:** 2021-09-14

Details of new titles for systematic reviews or evidence and gap maps that have been accepted by the Editor of a Campbell Coordinating Group are published in each issue of the journal. If you would like to receive a copy of the approved title registration form, please send an email to the Managing Editor of the relevant Coordinating Group.

## 1 CRIME AND JUSTICE

*Are tools that assess risk of violent radicalization and associated outcomes fit for purpose? A systematic review*

Ghayda Hassan, Sébastien Brouillette‐Alarie, Sarah Ousman, Jihan Rabah, Pablo Madriaza, Wynnpaul Varela, Emmanuel Danis, Deniz Kilinc, Loic Durocher‐Corfa, Mélina Girard, David Pickup, Eugene Borokhovski

10 May 2021

*Arts‐based interventions for offenders in secure criminal justice settings to improve rehabilitation outcomes: An evidence and gap map*

Jacqueline Tallent, Jes Phillips, Esther Coren

12 May 2021

*The effect of sports and physical activity interventions for at‐risk and offending children and youth on behavioural, psychosocial and offending outcomes: A mixed‐methods systematic review and meta‐analysis*

Suchi Malhotra, Ashima Mohan, Hannah Gaffney, Howard White

28 May 2021

## 2 EDUCATION

*School‐based social skills training for children with learning disabilities: A systematic review*

Kangle Guo, Xiuxia Li, Nan Chen, Yanfei Li, Lufang Feng, Jingwen Li, Jieyun Li, Xiajing Chu, Na Zhang, Howard White, Kehu Yang

27 May 2021

## 3 INTERNATIONAL DEVELOPMENT

*Measuring diet‐related consumer behaviours relevant to low‐ and middle‐income countries to advance food systems research: An evidence and gap map*

Ilse de Jager, Anne Sonneveld, Inge Brouwer, Eric Verger, Melissa Vargas, Ramani Wijesinha‐Bettoni, Ana Isla Ramos, Fatima Hachem

18 June 2021

## 4 METHODS

*Critical appraisal of methodological quality and reporting items of systematic reviews with meta‐analysis in evidence‐based social science in China: A systematic review*

Liping Guo, Wenjie Zhou, Xin Xing, Jieyun Li, Zhipeng Wei, Minyan Yang, Mina Ma, Howard White, Kehu Yang

6 July 2021

## 5 SOCIAL WELFARE

*Barriers and facilitators to accessing addictions treatment services for sexual minorities: A systematic review*

Miriam Hillyard, Beatrice Cockbain, Katharine Rimes, Colin Drummond

26 April 2021

*Interventions to increase physical activity among the older adults: An evidence and gap map*

Liujiao Cao, Xiuxia Li, Meixuan Li, Liangying Hou, Kehu Yang

SWCG

30 April 2021

*HIV prevention interventions for transgender women, transgender men, and cisgender men who have sex with men (TWSM, TMSM, and CMSM) in Latin America and the Caribbean: An evidence and gap map*

Rodrigo A. Aguayo‐Romero, Carlos Hermosa‐Bosano, Paula Hidalgo‐Andrade, and Ana María del Río‐González

3 May 2021

*An evidence and gap map of studies on digital interventions to reduce social isolation and loneliness among older adults*

Vivian Welch, Elizabeth Ghogomu, Victoria Barbeau, Rheda Adekpedjou, Sabrina Boutin, Niobe Haitas, Roger Simard, Paul Hebert, Chris Mikton

22 May 2021

*Intergenerational interventions and their effect on social and mental well‐being of both children and older people: A mapping review and evidence gap map*

Jo Thompson Coon, Fiona Campbell, Rebecca Whear, Jane Barlow, Ellie Robinson‐Carter, Morwenna Rogers, Anthea Sutton, Stuart Cohen, Richard Sharpe

28 May 2021

*Guaranteed basic income interventions for reducing poverty in high‐income countries: A systematic review*

Anita Rizvi, Marcia Gibson, Elizabeth Kristjansson, Christina Pollard, Vivian Welch, George A. Wells

14 June 2021

*Interventions aimed at stopping or reducing substance use in homeless adults*

Chris O'Leary, Rob Ralphs, Andrew Smith, Jordan Harrison, Zsolt Kiss

17 June 2021

*Global elder abuse: A mega‐map of systematic reviews on prevalence, consequences, risk and protective factors and interventions*

Christopher Mikton, Yongjie Yon, Marie Beaulieu; Kevin St‐Martin, Julien Cadieux Genesse, Jennifer Storey, Fiona Campbell, Michaela Rogers, Amanda Phelan, Mark Byrne, Parveen Ali, David Burnes, Bridget Penhale, Tova Band‐Winterstein, Mark Lachs, Karl Pillemer, Lilly Estenson, Kelly Marnfelt

30 June 2021

